# Strong Coupling Quantum Electrodynamics Hartree–Fock Response Theory

**DOI:** 10.1021/acs.jpca.5c01166

**Published:** 2025-05-09

**Authors:** Matteo Castagnola, Rosario R. Riso, Yassir El Moutaoukal, Enrico Ronca, Henrik Koch

**Affiliations:** † Department of Chemistry, 8018 Norwegian University of Science and Technology, 7491 Trondheim, Norway; ‡ Dipartimento di Chimica, Biologia e Biotecnologie, Università Degli Studi di Perugia, Via Elce di Sotto, 8, 06123 Perugia, Italy

## Abstract

The development of reliable *ab initio* methods for light-matter strong coupling is necessary for a deeper understanding of molecular polaritons. The recently developed strong coupling quantum electrodynamics Hartree–Fock model (SC-QED-HF) provides cavity-consistent molecular orbitals, overcoming several difficulties related to the simpler QED-HF wave function. In this paper, we further develop this method by implementing the response theory for SC-QED-HF. We compare the derived linear response equations with the time-dependent QED-HF theory and discuss the validity of equivalence relations connecting matter and electromagnetic observables. Our results show that electron-photon correlation induces an excitation redshift compared to the time-dependent QED-HF energies, and we discuss the effect of the dipole self-energy on the ground and excited state properties with different basis sets.

## Graphical abstract

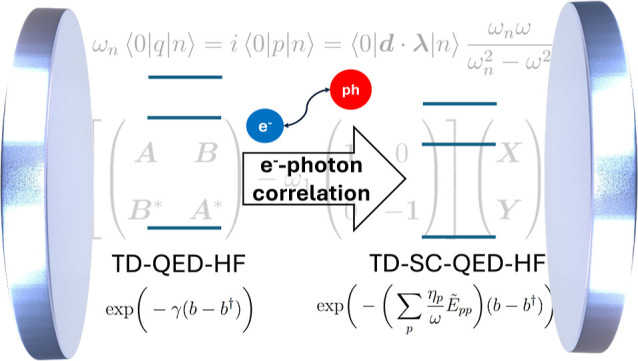

## 1 Introduction

Molecular polaritons are hybrid light-matter states arising from the interaction of electronic (or vibrational) excitations with the electromagnetic modes of an optical resonator.^1,2^ Chemists are currently trying to exploit polaritons to control and alter chemical processes, such as ground state and photochemical reactions.^3–7^ The theoretical description of these systems, in which both the molecular properties and the photon components play a central role, has led to a merging of quantum optics models and quantum chemistry approaches. The field of *ab initio* quantum electrodynamics (QED) is rapidly growing and several methods have been proposed, such as quantum electrodynamics Hartree–Fock (QED-HF),^8^ polarized Fock states,^9^ QED coupled cluster (QED-CC),^8,10–12^ QED full configuration interaction (QED-FCI),^8^ QED complete active space CI,^13^ and quantum electrodynamics density functional theory (QEDFT).^14,15^ *Ab initio* QED approaches describe the electronic structure (and the electron-photon coupling) at a high level of theory, although the computational complexity prevents the simulation of a large number of molecules. Light-matter strong coupling has so far been achieved in the collective regime, i.e., with a relatively small coupling strength and several ∼10^5^ molecules coupled to the same electromagnetic mode of an optical resonator. Nevertheless, experiments are pushing toward larger coupling strengths (ultrastrong coupling regime).^16^ The development of *ab initio* QED methods is relevant as it allows for a nonperturbative description of light-matter coupling while simultaneously providing reliable modeling of the molecular electronic structure. These methods also highlight subtleties in the description of the light-matter interplay,^17,18^ thus providing a deeper understanding of the polaritonic wave functions.

In this paper, we develop and implement linear response equations for the strong coupling quantum electrodynamics Hartree–Fock method (SC-QED-HF).^19,20^ The SC-QED-HF parametrization was introduced in 2022 by Riso et al.^19^ to solve issues in the Fock matrix of the QED-HF method, such as the origin-dependence of molecular orbitals for charged systems, and several other SC-QED-HF-like parametrizations have been proposed.^21,22^ The SC-QED-HF model mixes the electronic and photonic degrees of freedom and becomes exact in the infinite coupling limit, introducing to some extent what we refer to as “electron-photon correlation”.^8,19^ The SC-QED-HF wave function thus exhibits frequency dispersion (contrary to the QED-HF method), and the orbitals are consistent with increasing size of the system.^19,23^ A comparison of the QED-HF and SC-QED-HF results hence provides a simple way to assess the effect of electron-photon correlation on electronic and photonic properties.^19^ A set of reliable molecular orbitals is also of great assistance in developing post-HF polaritonic methodologies since part of the electron-photon correlation is already accounted for by the orbitals, improving the convergence and the quality of the simulation. Nevertheless, the changes in the electronic ground state induced by electronic strong coupling are usually small unless very large couplings are employed. In contrast, polaritonic excitations have a significant photon component (∼50%), and the Rabi splitting can reach a substantial fraction of the molecular excitation (ultrastrong coupling regime). Therefore, the self-consistent feedback between the electronic and electromagnetic degrees of freedom can be expected to have a more relevant effect than for the ground state. The developed response equations for SC-QED-HF then provide a straightforward way to assess the role of electron-photon correlation in the excited states and offer an additional step in developing consistent *ab initio* methods for molecular polaritons.

The paper is organized as follows. In Section 2, we introduce the Pauli-Fierz Hamiltonian and develop the QED-HF and SC-QED-HF ground state and response equations. We examine equivalence relations involving the transition moments for electronic and photonic observables and discuss the measure of the photon character of polaritonic excitations. In Section 3, we analyze the simulations of excited states and transition properties obtained from the SC-QED-HF response equations, highlighting the effects of electron-photon correlation and dipole self-energy in the excited states. Finally, in Section 4, we summarize our findings and discuss future perspectives for *ab initio* QED.

## 2 Theory

The electromagnetic and electronic degrees of freedom are treated at the same level of theory using the single-mode Pauli–Fierz Hamiltonian in the dipole approximation, Born–Oppenheimer approximation, and length form, here expressed in atomic units:^24–26^

1 $$H=\sum_{pq}h_{pq}E_{pq}+\frac{1}{2}\sum_{pqrs}g_{pqrs}e_{pqrs}+h_{nuc}+\frac{1}{2}\sum_{pqrs}{\left(\lambda \cdot d\right)}_{pq}{\left(\lambda \cdot d\right)}_{rs}E_{pq}E_{rs}-\sqrt{\frac{\omega }{2}}\sum_{pq}{\left(\lambda \cdot d\right)}_{pq}E_{pq}\left(b^{†}+b\right)+\omega b^{†}b$$

where *b* ^†^ (*b*) creates (annihilates) a photon of frequency ω, **λ** = λ**ϵ** is the light-matter coupling strength along the field polarization **ϵ**, *E*_*pq*_ and *e*_*pqrs*_ are the spin adapted one- and two-electron operators in a molecular basis indexed with *p*, *q*, *r*, and *s*.^27^ The ***d*** operator is the dipole operator, and *h*_*pq*_, *g*_*pqrs*_ and *h*_*nuc*_ are the one-electron integrals, two-electrons integrals, and nuclear repulsion, respectively. The Hamiltonian in eq 1 includes the standard electronic Hamiltonian (first line of eq 1), the energy of the photon field (last term), and the bilinear interaction (second to last term) between the molecular dipole and the photon field. The second line of eq 1 is the dipole self-energy, which is necessary for the Hamiltonian to have a ground state.^17,18^ In passing, we point out that in eq 1, we write the dipole self-energy contribution using the square of the dipole operator in second quantization. It is well-known that the product of first-quantization operators does not correspond to the product of the second-quantization operators unless a complete basis is employed.^27^ Alternatively, we can use the second quantization expression of the dipole self-energy from the quadrupole moment operator,^28,29^ which corresponds to a minor modification of the molecular integrals that is also implemented in the $e^{T}$ program.^30^ In this paper, all the calculations are performed using the Hamiltonian in eq 1.

The Hamiltonian in eq 1 serves as the foundation for an *ab initio* quantum electrodynamics treatment of the electron–photon system. The wave function parametrization must then include parameters for both the electronic and electromagnetic degrees of freedom. In the following section, we derive the ground state and response equations for QED-HF^8^ and SC-QED-HF.^19,20^

### 2.1 QED-HF and SC-QED-HF Ground State Parametrization

The QED-HF and SC-QED-HF wave functions are parametrized via a coherent state transformation *U*_X_ (with X = QED-HF or SC) applied to the tensor product of a single Slater determinant |S⟩ and the electromagnetic vacuum |0⟩:^8,19^

2 $$|GS\rangle =U_{X}\;(|S\rangle ⊗|0\rangle )$$

The state transformations *U*_X_ are given by

3 $$U_{QED‐HF}=\exp\left(-\gamma \left(b-b^{†}\right)\right)$$

4 $$U_{SC}=\exp\left(-\left(\sum_{p}\frac{\eta_{p}}{\omega }{Ẽ}_{pp}\right)\left(b-b^{†}\right)\right)$$

In eq 4, the parameters η_*p*_ are orbital-specific coherent state parameters associated with the orbital basis that diagonalizes the dipole interaction operator, indicated with a tilde:

5 $$\left(d\cdot \lambda \right)=\sum_{p}{\left(\mathrm{d̃}\cdot \lambda \right)}_{pp}{Ẽ}_{pp}$$

The orbital and photon parameters are obtained, using the variational principle, by minimizing the expectation value of the Hamiltonian in eq 1. In the following, the tilde spin-adapted operators ${Ẽ}_{pq}$ and ${ẽ}_{pqrs}$ will refer to the dipole basis of eq 5.

The optimal photon parameter γ of QED-HF in eq 3 is^8^

6 $$\gamma =\frac{\lambda \cdot {\left\langle d\right\rangle }_{\mathrm{QED‐HF}}}{\sqrt{2\omega }}$$

where ⟨ ***d*** ⟩_QED‑HF_ is the expectation value of the dipole operator for the optimal QED-HF Slater determinant. Transforming the Hamiltonian in eq 1 with the QED-HF operator *U*_QED‑HF_, we obtain

7 $$U_{\mathrm{QED‐HF}}^{†}HU_{\mathrm{QED‐HF}}=\sum_{pq}h_{pq}E_{pq}+\frac{1}{2}\sum_{pqrs}g_{pqrs}e_{pqrs}+h_{nuc}+\frac{1}{2}\sum_{pqrs}{\left(\lambda \cdot \left(d-{\left\langle d\right\rangle }_{QED‐HF}\right)\right)}_{pq}{\left(\lambda \cdot \left(d-{\left\langle d\right\rangle }_{\mathrm{QED‐HF}}\right)\right)}_{rs}E_{pq}E_{rs}-\sqrt{\frac{\omega }{2}}\sum_{pq}{\left(\lambda \cdot \left(d-{\left\langle d\right\rangle }_{\mathrm{QED‐HF}}\right)\right)}_{pq}E_{pq}\left(b+b^{†}\right)+\omega b^{†}b$$

which is manifestly origin invariant even for charged systems. The molecular orbitals are obtained from the QED Fock matrix^8^

8 $$F_{pq}=F_{pq}^{e}+\frac{1}{2}\left(\sum_{a}\left(\lambda \cdot d_{pa}\right)\left(\lambda \cdot d_{aq}\right)-\sum_{i}\left(\lambda \cdot d_{pi}\right)\left(\lambda \cdot d_{iq}\right)\right)$$

where *F*_*pq*_^*e*^ is the standard electronic Fock matrix.^27^ In eq 8, indexes *i* and *a* refer to occupied and virtual orbitals, respectively. The occupied and virtual blocks of the Fock matrix in eq 8 are origin-dependent for charged systems. Therefore, the molecular orbitals change accordingly (although the total energy is origin invariant).

The SC-QED-HF parametrization in eq 4 is introduced to obtain more consistent molecular orbitals.^19,20^ The transformation *U*_SC_ explicitly correlates electronic and photonic degrees of freedom: the electronic creation operators ${ã}_{p}^{†}$ , when transformed with *U*_SC_, reads

9 $$U_{SC}^{†}\;{ã}_{p}^{†}\;U_{SC}={ã}_{p}^{†}\;\exp\left(\frac{\eta_{p}}{\omega }\left(b-b^{†}\right)\right)$$

That is, each electronic creation (annihilation) operator in the dipole basis is dressed with a coherent state of the photon field, and the SC-transformed Hamiltonian reads

10 $$H_{SC}=U_{SC}^{†}HU_{SC}=\sum_{pq}{\mathrm{h̃}}_{pq}{Ẽ}_{pq}\;\exp\left(\frac{1}{\omega }\left(\eta_{p}-\eta_{q}\right)\left(b-b^{†}\right)\right)+\frac{1}{2}\sum_{pqrs}{\mathrm{g̃}}_{pqrs}{ẽ}_{pqrs}\;\exp\left(\frac{1}{\omega }\left(\eta_{p}+\eta_{r}-\eta_{q}-\eta_{s}\right)\left(b-b^{†}\right)\right)+h_{nuc}+\omega \left(b^{†}-\sum_{p}\left(\frac{\lambda }{\sqrt{2\omega }}{\left(\mathrm{d̃}\cdot \epsilon \right)}_{pp}-\frac{\eta_{p}}{\omega }\right){Ẽ}_{pp}\right)\;\left(b-\sum_{p}\left(\frac{\lambda }{\sqrt{2\omega }}{\left(\mathrm{d̃}\cdot \epsilon \right)}_{pp}-\frac{\eta_{p}}{\omega }\right){Ẽ}_{pp}\right)$$

The electromagnetic and electronic degrees of freedom are thus entangled via the transformation in eq 4, and simultaneous optimization of the η_*p*_ and orbital parameters is required.^20^ We notice that, from eq 4, we can recover the QED-HF parametrization by setting η_*p*_ = ωγ/*N*, where *N* is the number of electrons in the system, and therefore the SC-QED-HF variational energy is always lower than the QED-HF energy.

The SC-QED-HF model provides a significant improvement over the simpler QED-HF wave function. First, the SC-QED-HF parametrization solves the origin dependence issues for charged systems. Second, the wave function shows dispersive behavior with the cavity frequency, contrary to the QED-HF energy and orbitals, which are ω-independent.^19,20^ Moreover, the QED-HF orbitals show a troublesome behavior when two subsystems are separated over large distances, which can be particularly problematic when addressing perturbation theory or the collective coupling regime.^23^ A set of QED-consistent molecular orbitals is also relevant for the development of reliable post-HF methods.

#### 2.1.1 Infinite Coupling Limit and Electron-Photon Correlation

The SC-QED-HF parametrization is introduced from the infinite coupling limit *H*_∞_ of the dipole Hamiltonian:^19,31,32^

11 $$H_{\infty }=\omega b^{†}b-\lambda \sqrt{\frac{\omega }{2}}\left(d\cdot \epsilon \right)\left(b^{†}+b\right)+\frac{\lambda^{2}}{2}{\left(d\cdot \epsilon \right)}^{2}$$

If we set $\eta_{p}=\sqrt{\frac{\omega }{2}}{\left(\mathrm{d̃}\cdot \lambda \right)}_{pp}$ in *U*_SC_ and transform *H*_∞_, we find that the transformation diagonalizes the Hamiltonian in eq 11. Therefore, as the coupling increases, the SC-QED-HF model approaches the exact solution in which the electron and photon degrees of freedom are deeply entangled (see eq 9).^19,31,32^ On the other hand, the QED-HF orbitals also approach the dipole basis, but *U*_QED‑HF_ fails to properly correlate the electrons and the photon field.

In the HF ansatz, the electrons are treated independently from one another (except for the Fermi correlation arising from the wave function antisymmetry). The instantaneous electronic position is thus not relevant since each electron perceives a mean-field Coulomb effect from the electrons in the other occupied orbitals. Therefore, the HF method is considered uncorrelated. In electronic structure theory, electron–electron correlation is then defined as the difference between the full configuration-interaction (FCI) energy and the HF energy, within the employed basis set.^27^ The same definition is used for the correlation energy in approximate methods such as coupled cluster or truncated CI. In the *ab initio* QED framework, we introduce the photons as additional boson particles. Therefore, electron correlation is modified, e.g., by additional photon-mediated electron interactions (electron–photon–electron interactions). The entanglement of the electromagnetic and electronic degrees of freedom requires a definition of electron-photon correlation. Since the electronic and electromagnetic degrees of freedom are deeply intertwined, it can be cumbersome to separate electronic and electron-photon effects, especially for large λ and highly correlated methods. However, SC-QED-HF becomes exact in the infinite coupling limit (where electron-photon correlation dominates over any electronic effect), and the comparison between the mean field QED-HF and the entangled SC-QED-HF wave function provides a simple measure of the electron-photon correlation in the ground state. For the excited states, it is even harder to provide an effective definition of electron-photon correlation since matter and photon excitations have a similar weight in the polaritonic states. We will nevertheless argue that SC-QED-HF response theory can lead to a simple understanding of excited state electron-photon correlation.

### 2.2 Response Theory for SC-QED-HF

The QED-HF and SC-QED-HF response theory is based on the TD-QED-HF parametrization:^25^

12 $$|R\left(t\right)\rangle =\exp\left(-i\Lambda \left(t\right)\right)|S,0\rangle =\exp\left(-i\kappa \left(t\right)\right)\exp\left(-i\left(\gamma \left(t\right)b^{†}+\gamma^{*}\left(t\right)b\right)\right)|R\rangle$$

where |R⟩ = |S⟩⊗|0⟩ is the optimal determinant in the electromagnetic vacuum, γ describes the field evolution, and **κ**(*t*) is an orbital rotation operator:

13 $$\kappa \left(t\right)=\frac{1}{\sqrt{2}}\sum_{ai}\left(\kappa_{ai}E_{ai}+\kappa_{ai}^{*}E_{ia}\right)$$

where *i* (*a*) label occupied (virtual) orbitals of the reference determinant. In eq 12, |S⟩ is the reference (SC-)­QED-HF Slater determinant |(SC-)­QED-HF⟩, and the parametrization refers to the transformed Hamiltonian

14 $$H_{X}=U_{X}^{†}HU_{X}$$

with X = QED-HF or SC. That is, the TD-QED-HF and TD-SC-QED-HF wave functions refer respectively to the Hamiltonians in eqs 7 and 10. The transformation *U*_SC_ ensures that for the infinite coupling limit in eq 11, the parametrization in eq 12 recovers the exact solutions. The response equations for the Hamiltonian *U*_X_^†^(*H* + *V*)*U*_X_, where *V* is an external perturbation operator, are obtained from a perturbation expansion in the frequency domain:^33^

15 $$\Lambda \left(t\right)=\Lambda^{\left(1\right)}\left(t\right)+\Lambda^{\left(2\right)}\left(t\right)+...=\int d\omega_{1}e^{-i\omega_{1}t}\Lambda^{\omega_{1}}+\frac{1}{2}\iint d\omega_{1}d\omega_{2}e^{-i\omega_{1}t}e^{-i\omega_{2}t}\Lambda^{\omega_{1},\omega_{2}}+...$$

where

16 $$\Lambda^{\omega_{1}}=\frac{1}{\sqrt{2}}\sum_{ai}\left(\kappa_{ai}^{\omega_{1}}E_{ai}+{\left[\kappa_{ai}^{-\omega_{1}}\right]}^{*}E_{ia}\right)+\left(\gamma^{\omega_{1}}b^{†}+{\left[\gamma^{-\omega_{1}}\right]}^{*}b\right)$$

17 $$\Lambda^{\omega_{1},\omega_{2}}=\frac{1}{\sqrt{2}}\sum_{ai}\left(\kappa_{ai}^{\omega_{1},\omega_{2}}E_{ai}+{\left[\kappa_{ai}^{-\omega_{1},-\omega_{2}}\right]}^{*}E_{ia}\right)+\left(\gamma^{\omega_{1},\omega_{2}}b^{†}+{\left[\gamma^{-\omega_{1},-\omega_{2}}\right]}^{*}b\right)$$

Using eq 15, we define the response functions from the expectation value of an operator *A*:

18 $$\left\langle A\right\rangle \left(t\right)={\left\langle A\right\rangle }_{R}+\int d\omega_{1}e^{-i\omega_{1}t}{\left\langle \left\langle A;V^{\omega_{1}}\right\rangle \right\rangle }_{\omega_{1}}+\frac{1}{2}\iint d\omega_{1}d\omega_{2}e^{-i\omega_{1}t}e^{-i\omega_{2}t}{\left\langle \left\langle A;V^{\omega_{1}},V^{\omega_{2}}\right\rangle \right\rangle }_{\omega_{1},\omega_{2}}+...$$

The linear response equations, which can be obtained following the same derivation as Olsen and Jørgensen based on Eherenfest’s theorem,^33^ are the same for the two parametrizations:

19 $$\left[\left(\begin{array}{cc} A & B\\ B^{*} & A^{*} \end{array}\right)-\omega_{1}\left(\begin{array}{cc} 1 & 0\\ 0 & -1 \end{array}\right)\right]\left(\begin{array}{l} X\\ Y \end{array}\right)=i\left(\begin{array}{l} g_{1}\\ g_{2} \end{array}\right)\equiv ig$$

where the vectors ***X*** and ***Y*** collect the Fourier transformed parameters:

20 $$X=\left(\begin{array}{l} \gamma^{\omega_{1}}\\ \kappa_{ai}^{\omega_{1}} \end{array}\right),Y=\left(\begin{array}{l} {\left[\gamma^{-\omega_{1}}\right]}^{*}\\ {\left[\kappa_{ai}^{-\omega_{1}}\right]}^{*} \end{array}\right)$$

The right-hand side of eq 19 is the generalized gradient

21 $$g_{1}=\left(\begin{array}{c} {\left\langle \left[b,V_{X}^{\omega_{1}}\right]\right\rangle }_{R}\\ \frac{1}{\sqrt{2}}{\left\langle \left[E_{ia},V_{X}^{\omega_{1}}\right]\right\rangle }_{R} \end{array}\right),g_{2}=\left(\begin{array}{c} {\left\langle \left[b^{†},V_{X}^{\omega_{1}}\right]\right\rangle }_{R}\\ \frac{1}{\sqrt{2}}{\left\langle \left[E_{ai},V_{X}^{\omega_{1}}\right]\right\rangle }_{R} \end{array}\right)$$

and the explicit expressions of ***A*** and ***B*** are

22 $$A=\left(\begin{array}{cc} {\left\langle \left[b,\left[H_{X},b^{†}\right]\right]\right\rangle }_{R} & \frac{1}{\sqrt{2}}{\left\langle \left[b,\left[H_{X},E_{ai}\right]\right]\right\rangle }_{R}\\ \frac{1}{\sqrt{2}}{\left\langle \left[b^{†},\left[H_{X},E_{ia}\right]\right]\right\rangle }_{R} & \frac{1}{2}{\left\langle \left[E_{jb},\left[H_{X},E_{ai}\right]\right]\right\rangle }_{R} \end{array}\right)$$

23 $$B=\left(\begin{array}{cc} {\left\langle \left[b,\left[H_{X},b\right]\right]\right\rangle }_{R} & \frac{1}{\sqrt{2}}{\left\langle \left[b,\left[H_{X},E_{ia}\right]\right]\right\rangle }_{R}\\ \frac{1}{\sqrt{2}}{\left\langle \left[b^{†},\left[H_{X},E_{ai}\right]\right]\right\rangle }_{R} & \frac{1}{2}{\left\langle \left[E_{bj},\left[H_{X},E_{ai}\right]\right]\right\rangle }_{R} \end{array}\right)$$

Therefore, eq 19 is a generalization of the Casida equations of TDHF in molecular response theory, including additional parameters describing the photon field.^25,34–36^ The difference between the QED-HF and SC-QED-HF response equations is embedded in the picture change *U*_X_ and the orbitals of the reference determinant |S⟩. The computational cost of the two response methods is ∼*M* ^4^ where *M* is the number of basis functions,^37^ though SC-QED-HF involves the calculation of the two-electron integrals in the dipole basis, which can be done efficiently with Cholesky decomposed two-electron integral.^20^ Since the SC-QED-HF model introduces electron-photon correlation via the *U*_SC_ transformation, a comparison between TD-QED-HF and TD-SC-QED-HF reveals the impact of electron-photon correlation on the excited states.

#### 2.2.1 Equivalence Relations

The exact linear response functions fulfill the equation of motion^33^

24 $$\omega_{1}{\left\langle \left\langle A;V^{\omega_{1}}\right\rangle \right\rangle }_{\omega_{1}}={\left\langle \left\langle \left[A,H\right];V^{\omega_{1}}\right\rangle \right\rangle }_{\omega_{1}}+\left\langle 0\left|\left[A,V^{\omega_{1}}\right]\right|0\right\rangle$$

From the position-momentum relation,

25 $$ip_{i}=\left[r_{i},H\right]$$

which also holds for the dipole Hamiltonian in length form in eq 1, we obtain the equivalence between the dipole and velocity formulation of the transition moments:

26 $$\omega_{n}\left\langle 0\left|r_{i}\right|n\right\rangle =i\left\langle 0\left|p_{i}\right|n\right\rangle$$

where ω_*n*_ is the excitation energy from the ground state to the excited state |*n*⟩.

Analogous equivalence relations can be derived for photon observables.^25^ In fact, an equation analogous to eq 25 also holds for the photon coordinate $q=\frac{1}{\sqrt{2\omega }}\left(b+b^{†}\right)$ and photon momentum $p=i\sqrt{\frac{\omega }{2}}\left(b^{†}-b\right)$ for the Hamiltonian in eq 1

27 ip=[q,H]

which provides a relation between the corresponding transition moments:

28 $$\omega_{n}\left\langle 0\left|q\right|n\right\rangle =i\left\langle 0\left|p\right|n\right\rangle$$

It is also possible to derive relations that connect photon and electronic quantities. For the operators *b* and *b* ^†^, we can obtain the identities

29 $$\omega_{1}{\left\langle \left\langle b;V^{\omega_{1}}\right\rangle \right\rangle }_{\omega_{1}}={\left\langle \left\langle -\sqrt{\frac{\omega }{2}}d\cdot \lambda -\omega b;V^{\omega_{1}}\right\rangle \right\rangle }_{\omega_{1}}+\left\langle 0\left|\left[b,V^{\omega_{1}}\right]\right|0\right\rangle$$

30 $$\omega_{1}{\left\langle \left\langle b^{†};V^{\omega_{1}}\right\rangle \right\rangle }_{\omega_{1}}={\left\langle \left\langle \sqrt{\frac{\omega }{2}}d\cdot \lambda +\omega b^{†};V^{\omega_{1}}\right\rangle \right\rangle }_{\omega_{1}}+\left\langle 0\left|\left[b^{†},V^{\omega_{1}}\right]\right|0\right\rangle$$

from which we obtain^25^

31 $$\omega_{n}\left\langle 0\left|q\right|n\right\rangle =i\left\langle 0\left|p\right|n\right\rangle =\left\langle 0\left|d\cdot \lambda \right|n\right\rangle \frac{\omega_{n}\omega }{\omega_{n}^{2}-\omega^{2}}$$

The relation in eq 31 relates electromagnetic and molecular observables, reflecting the intrinsic connection of the electronic and photonic degrees of freedom.

These equivalence relations can have relevant physical meanings and can be of practical importance in simulations. For instance, from the quadratic response function, it is possible to show that $\left\langle n\left|{Ê}_{⊥}\right|n\right\rangle =0$ , where ***Ê***_⊥_ = −**λ**(**λ**· ***d***) – **λ**ω*q* is the electric field operator, which guarantees that the ground state is nonradiating. At the same time, eq 26 is fundamental for obtaining origin invariant optical rotational strengths.^38^ Although relevant, these equivalence relations are not guaranteed for approximate wave functions described in a finite basis set. In particular, the relation in eq 25 requires basis set completeness,^27^ while eq 27 does not depend on the basis set size. In ref 25, it was shown that the relations eqs 26 and 31 hold for TD-QED-HF. In particular, while eq 26 holds only for a complete basis set, eq 31 is fulfilled independent of basis set size.[Fn fn1] For the SC-QED-HF wave function, the *U*_SC_ transformation mixes electronic and photonic operators,

32 $$U_{SC}^{†}{Ẽ}_{pq}U_{SC}={Ẽ}_{pq}\;\exp\left(\frac{\eta_{p}-\eta_{q}}{\omega }\left(b-b^{†}\right)\right)$$

33 $$U_{SC}^{†}bU_{SC}=b+\sum_{p}\frac{\eta_{p}}{\omega }{Ẽ}_{pp}$$

which complicates the analysis. However, since the transformation *U*_SC_ commutes with the photon momentum *p* and the dipole operator ***d***, it is possible to show that the relations in eq 26 (for a complete basis set) and eq 31 are fulfilled for the proposed TD-SC-QED-HF model (see the Supporting Information).

#### 2.2.2 Photon Character and Picture Change

Polaritons are states of hybrid light-matter character, and the photon component plays a relevant role in their properties. It is then interesting to discuss the relative contribution of the photon and matter excitations in the polaritonic wave function. In this section, we highlight the difficulties encountered in providing a clear definition of the “photon character” of excitations.

The response parameter γ, associated with the photon operator *b* ^†^ in eq 16, can provide a measure of the photon weight ϑ_*n*_ in the excited state |*n*⟩:^39^

34 $$\vartheta_{n}=\gamma_{n}^{2}$$

Nevertheless, care is advised in adopting such an interpretation since the analysis is hindered by the picture change *U*_X_ in eq 14, especially for SC-QED-HF as *U*_SC_ mixes electronic and photonic components. Moreover, the Hamiltonian in eq 1 originates from the Power–Zienau–Woolley (PZW) transformation of the minimal coupling Hamiltonian (within the dipole approximation).^24,26^ As a consequence, the photon coordinate $q=\frac{1}{\sqrt{2\omega }}\left(b+b^{†}\right)$ is connected to the displacement field ***D*** of the macroscopic Maxwell’s equations rather than the microscopic electric field ***E***.^17,24–26^ Since the electronic and electromagnetic degrees of freedom are intertwined, it is then questionable to think of *b* ^†^ as a “purely photonic” operator. In addition, the photon component of the ground state is nonnegligible since we describe the electron-photon interaction non-perturbatively within an *ab initio* framework. Our ground state results thus differ from the simplified Jaynes–Cummings model,^40^ where the rotating wave approximation and the neglect of the dipole self-energy results in an unmodified electronic ground state in the electromagnetic vacuum. It is accordingly difficult to provide a clear definition of the “photon character” of excitations, and interpretations of such quantities can be misleading, especially for large coupling strengths λ.

Photonic observables can provide a more rigorous measure of the photon role in the wave function. The photon count *b* ^†^ *b* is a natural choice, though its representation changes with *U*_X_ and it is thus generally nonzero for the ground state. Moreover, the photon number operator *b* ^†^ *b* for the length Hamiltonian does not correspond to the photon number operator in the velocity form. The mathematical expression for photon number *b* ^†^ *b* in the length gauge representation (eq 1) transformed to the (SC-)­QED-HF picture in eq 7 reads

35 $$b^{†}b\to {U_{X}}^{†}b^{†}bU_{X}=\{\begin{array}{ll} \left(b^{†}+\frac{\lambda \cdot {\left\langle d\right\rangle }_{QED‐HF}}{\sqrt{2\omega }}\right)\;\left(b+\frac{\lambda \cdot {\left\langle d\right\rangle }_{QED‐HF}}{\sqrt{2\omega }}\right) & QED‐HF\\ \left(b^{†}+\sum_{p}\frac{\eta_{p}}{\omega }{Ẽ}_{pp}\right)\;\left(b+\sum_{p}\frac{\eta_{p}}{\omega }{Ẽ}_{pp}\right) & SC‐QED‐HF \end{array}$$

These expressions should be used to obtain the photon count in the ground and excited states.^28,41^ The operator expression for photon number in the velocity-representation is, in the (SC-)­QED-HF picture, similarly obtained from the PZW transformation *U*_PZW_:^28,41^

36 $${b^{†}b\to U}_{X}^{†}U_{PZW}^{†}b^{†}bU_{PZW}U_{X}=\begin{array}{l} \{\begin{array}{ll} \left(b^{†}-\frac{\lambda \cdot \left(d-{\left\langle d\right\rangle }_{QED‐HF}\right)}{\sqrt{2\omega }}\right)\;\left(b-\frac{\lambda \cdot \left(d-{\left\langle d\right\rangle }_{QED‐HF}\right)}{\sqrt{2\omega }}\right) & QED‐HF\\ \left(b^{†}-\left(\frac{\lambda \cdot d}{\sqrt{2\omega }}-\sum_{p}\frac{\eta_{p}}{\omega }{Ẽ}_{pp}\right)\right)\;\left(b-\left(\frac{\lambda \cdot d}{\sqrt{2\omega }}-\sum_{p}\frac{\eta_{p}}{\omega }{Ẽ}_{pp}\right)\right) & SC‐QED‐HF \end{array} \end{array}$$

In general, using these operators will provide different results,^9,17,41^ and we do not expect any of these operators to have zero expectation value for the ground or the excited states. Computing ⟨*n*|*b* ^†^ *b*|*n*⟩ (or the corresponding quantities for the SC and velocity representations) further requires the quadratic response functions once the excitation energy ω_*n*0_ and the transition moments from the ground |0⟩ to the excited state |*n*⟩ (as well as the ground state expectation value ⟨0|*A*|0⟩), have been computed:^33^

37 $$\lim_{\omega_{1}\to \omega_{n0}}\left(\omega_{n0}+\omega_{1}\right)\;\lim_{\omega_{2}\to \omega_{n0}}\left(\omega_{n0}-\omega_{2}\right)\;{\left\langle \left\langle A;V^{\omega_{1}},V^{\omega_{2}}\right\rangle \right\rangle }_{\omega_{1},\omega_{2}}=\left\langle 0\left|V^{\omega_{1}}\right|n\right\rangle \left(\left\langle n\left|A\right|n\right\rangle -\left\langle 0\left|A\right|0\right\rangle \right)\left\langle n\left|V^{\omega_{2}}\right|0\right\rangle$$

Therefore, we suggest a different quantity as a measure of the photon character of a polaritonic excitation that can be complementary to eq 34. The photon momentum $ip=\sqrt{\frac{\omega }{2}}\left(b-b^{†}\right)$ commutes with the operator *U*_*X*_ and is connected to the vector potential operator in the velocity representation. The expectation value ⟨*n*|*p*|*n*⟩ (which would, in principle, require quadratic response functions) is identically zero for any real wave function. Using the operator *p* is thus convenient since its expectation value vanishes identically for the (SC-)­QED-HF ground state and has a clear physical interpretation. Since ⟨*n*|*p*|*n*⟩ = 0, we rely on the transition moments |⟨R|*p*|*n*⟩|^2^, which can be computed from the linear response equations, as an indication of how the photon component changes following a transition from the ground state |R⟩ to the excited state |*n*⟩.

## 3 Results

In this section, we present the results for the TD-SC-QED-HF linear response method outlined in the previous section, also providing a comparison with the TD-QED-HF model. The QED-HF, SC-QED-HF, and TD-QED-HF equations are implemented in the development branch of the $e^{T}$ program, an open-source electronic structure program.^30^ The TD-SC-QED-HF model has been implemented in a local branch of the $e^{T}$ program.

### 3.1 Equivalence Relations and Dipole Self-Energy

In Table 1, we report transition observables computed using different basis sets for the lower polariton (LP) of a formaldehyde molecule in an optical cavity with field polarization parallel to the transition dipole of the first bright molecular excitation. The oscillator strengths in velocity *f*_*v*_ and length *f*_*l*_ forms

38 $$f_{l}=\frac{2}{3}\omega_{LP}{\left|\left\langle GS\left|d\right|LP\right\rangle \right|}^{2},f_{v}=\frac{2}{3}\frac{1}{\omega_{LP}}{\left|\left\langle GS\left|p\right|LP\right\rangle \right|}^{2}$$

where ω_*LP*_ is the LP excitation energy, are connected to the intensity of the electronic excitation. Based on the equivalence relation in eq 26, the velocity and length gauge oscillator strengths should converge when approaching basis set completeness. In Table 1, we also report the transition moments for the photon coordinate *q* and momentum *p*, and focus on the equivalence relation in eq 31. While the commutator relation in eq 25 requires a complete basis set to be fulfilled,^27^ eq 27 is basis-set independent. Since the optimization of the SC-QED-HF wave function is not performed within a truncated photon subspace, the photonic equivalence relations in eq 31 are fulfilled exactly for any basis set size for TD-SC-QED-HF, similarly to the TD-QED-HF model.^25^ If we rely on the Tamm-Dancoff approximation (TDA) by disregarding the ***B*** block in the response equations in eq 19 (which is equivalent to a CIS model), the relations in eq 26 and eq 28 are not guaranteed anymore.

**Table 1:** Oscillator Strengths (in Length Gauge $f_{l}=\frac{2}{3}\omega_{LP}{\left|\left\langle GS\left|d\right|LP\right\rangle \right|}^{2}$ and Velocity Gauge $f_{v}=\frac{2}{3}\frac{1}{\omega_{LP}}{\left|\left\langle GS\left|p\right|LP\right\rangle \right|}^{2}$ ) and Transition Moments for the Photon Coordinate $q=\frac{1}{\sqrt{2\omega }}\left(b+b^{†}\right)$ and Photon Momentum $ip=\sqrt{\frac{\omega }{2}}\left(b-b^{†}\right)$ for the First Polaritonic State (LP) of Formaldehyde Computed Using TD-SC-QED-HF in Different Basis Sets^a^

basis	*f*_*l*_	*f*_*v*_	*q*_0*n*_ [a.u.]	*ip*_0*n*_ [a.u.]	ω_*n*_ *q*_0*n*_ [a.u.]	$\frac{\omega \omega_{n}{\left(\lambda \cdot d\right)}_{0n}}{\omega_{n}^{2}-\omega^{2}}\;[\mathrm{a.u.}]$
sto-3g	0.0001	0.0001	1.26635123	0.39477749	0.39477749	0.39477749
6-31g	0.0014	0.0011	1.26577915	0.39432973	0.39432973	0.39432973
6-31g*	0.0015	0.0013	1.26582131	0.39432455	0.39432455	0.39432455
6-31g**	0.0016	0.0014	1.26580336	0.39431348	0.39431348	0.39431348
6-31+g**	0.0042	0.0041	1.25502739	0.39067397	0.39067397	0.39067397
6-31++g**	0.0151	0.0140	1.15402184	0.35853984	0.35853984	0.35853984
aug-cc-pvdz	0.0222	0.0219	1.01273656	0.31418802	0.31418802	0.31418802
d-aug-cc-pvdz	0.0258	0.0252	0.88685195	0.27480100	0.27480100	0.27480100
d-aug-cc-pvtz	0.0203	0.0202	1.03445988	0.32103706	0.32103706	0.32103706

a The photon frequency is set to ω = 0.311860 a.u., the coupling strength is λ = 0.01 a.u., and the molecular geometry is reported in the Supporting Information. The photon field is perpendicular to the C–O bond in the molecular plane. In the table, we also report the photon and electron–photon quantities shown in eq 31. While the position-momentum equivalence in eq 26 is only fulfilled for a complete basis set, the relation in eq 31 is independent of the basis set choice.

For the calculations reported in Table 1, we used the Hamiltonian in eq 1, which includes the dipole self-energy (DSE). The DSE ensures the Hamiltonian to be bounded from below, and thus, the system has a stable ground state.^17,18^ If the DSE is disregarded, the energy of the system can become infinitely low by displacing the electrons far away from the nuclear core along the polarization direction. However, since the atomic orbitals are centered on the nuclei, such electronic displacement is generally hard to produce within a finite basis set. It is thus worth investigating how the molecular properties behave when the basis set is increased without having the DSE in the Hamiltonian. In Table 2, we report the ground state energy, the LP excitation energy, and transition properties for a formaldehyde molecule, computed including or disregarding the DSE in the Pauli-Fierz Hamiltonian. The difference in the computed ground state energy is of the order of ∼10^–4^ a.u., which, for λ = 0.01 a.u., is consistent with a perturbative analysis of the DSE. However, we see that the energy difference does increase with the basis set size. A similar trend is observed for the excitation energy ω_*LP*_. For the transition properties and large basis sets, we notice a significant difference in the photon transition properties *q*_0*n*_ and *ip*_0*n*_ and an appreciable difference in the oscillator strength *f*_*l*_. We emphasize that formaldehyde is a small molecule, and these effects are expected to increase with system size and coupling strength λ. In addition, the DSE ensures gauge invariance and guarantees the Hamiltonian behaves correctly for large coupling strengths.^17–19^ Since the effect of the DSE on the transition properties can be non-negligible, we believe including the DSE in the Hamiltonian is preferable, although we do not observe large qualitative changes in the ground state results.

**Table 2:** Ground State (GS) Energy, Lower Polariton (LP) Excitation Energy, Length-Gauge Oscillator Strength *f*_*l*_, and Photon Transition Moments *q*_0*n*_ and *ip*_0*n*_ for a Formaldehyde Molecule in Different Basis Set with and without the Dipole Self-Energy (DSE) Term in the Hamiltonian^a^

basis	*E*_GS_ [a.u.]	ω_*LP*_ [a.u.]	*f*_*l*_	*ip*_0*n*_ [a.u.]	*q*_0*n*_ [a.u.]
TD-SC-QED-HF/Pauli–Fierz Hamiltonian					
sto-3g	–112.35337118	0.311744	0.0001	1.26635123	0.39477749
6-31g	–113.80630521	0.311531	0.0014	1.26577915	0.39432973
6-31g*	–113.86418524	0.311516	0.0015	1.26582131	0.39432455
6-31g**	–113.86774654	0.311512	0.0016	1.26580336	0.39431348
6-31+g**	–113.87256586	0.311287	0.0042	1.25502739	0.39067397
6-31++g**	–113.87273099	0.310687	0.0151	1.15402184	0.35853984
aug-cc-pvdz	–113.88439824	0.310236	0.0222	1.01273656	0.31418802
d-aug-cc-pvdz	–113.88476414	0.309861	0.0258	0.88685195	0.27480100
d-aug-cc-pvtz	–113.91293118	0.310342	0.0203	1.03445988	0.32103706
TD-SC-QED-HF/no dipole self-energy (DSE)					
sto-3g	–112.35351682	0.311743	0.0001	1.26635126	0.39477740
6-31g	–113.80653092	0.311530	0.0014	1.26577394	0.39432692
6-31g*	–113.86449828	0.311515	0.0015	1.26581618	0.39432172
6-31g**	–113.86806291	0.311511	0.0016	1.26579796	0.39431052
6-31+g**	–113.87288665	0.311278	0.0044	1.25431979	0.39044273
6-31++g**	–113.87305235	0.310558	0.0176	1.12292261	0.34873366
aug-cc-pvdz	–113.88475188	0.309964	0.0257	0.93366699	0.28940407
d-aug-cc-pvdz	–113.88512056	0.309456	0.0287	0.77936226	0.24117891
d-aug-cc-pvtz	–113.91328959	0.310065	0.0238	0.94848608	0.29409292

a The photon field is aligned perpendicular to the C–O bond in the molecular plane. The photon frequency is set to ω = 0.311860 a.u., the coupling strength is λ = 0.01 a.u., and the molecular geometry is reported in the Supporting Information.

### 3.2 Effects of Electron-Photon Correlation on the Excited States

In Table 3, we report the ground state energy *E*_GS_, the LP excitation energy ω_*LP*_, the length gauge oscillator strength *f*_*l*_, the photon character ϑ_*LP*_ in eq 34, and the photon momentum and coordinate transition moments for the *p*-nitroaniline (PNA) in the aug-cc-pVDZ basis set, computed using the TD-QED-HF and TD-SC-QED-HF models for different coupling strengths λ (the upper polariton data are reported in the Supporting Information). The employed coupling constants correspond to the following quantization volumes: *V* ≈ 74.4, 18.6, 3.0 and 0.7 nm^3^. In experimental setups, light-matter strong coupling is achieved via a collective coupling, with several (∼10^5^ to 10^7^) molecules interacting with the same optical mode. Some of the employed single-molecule couplings are unrealistic for current experimental devices, and it is still unclear if and to what extent a single-molecule calculation with artificially large coupling can reproduce the effect of collective strong coupling.^42–47^ Nevertheless, we are here interested in the effect of electron-photon correlation on the wave function parametrization rather than in a careful comparison with experimental results. The use of large single-molecule coupling is thus not a limitation for this study, and we will later address the effect of the collective coupling. In Table 3, we notice that the photon character ϑ_*LP*_ increases with the coupling strength, following the same trend as the oscillator strength. This counterintuitive trend (the photon states carry zero oscillator strength) was already emphasized in ref 39 and was explained with the intrusion of higher energy states in the excitation, which compensate for the larger photon character.^39^ Here, we notice that the transition photon coordinate ⟨GS|*q*|LP⟩ and momentum ⟨GS|*ip*|LP⟩ also follow the same trend. The increased excitation strength can then be rationalized via eq 31

39 $$\left\langle 0\left|d\cdot \lambda \right|n\right\rangle =\frac{\omega_{n}^{2}-\omega^{2}}{\omega }\left\langle 0\left|q\right|n\right\rangle =i\frac{\omega_{n}^{2}-\omega^{2}}{\omega_{n}\omega }\left\langle 0\left|p\right|n\right\rangle$$

from which we notice that the transition moment along the polarization direction increases with the transition photon moments and with the Rabi splitting. In Table 3, we also notice that the TD-SC-QED-HF excitation energies are lower than the TD-QED-HF energies. Since the two models have the same response parametrization and zero-coupling limit, we can rationalize this result in terms of electron-photon correlation. As explained in Sec. [Sec sec2], the SC-QED-HF ground state energy is always lower than the QED-HF energy, as can be verified in Table 3. This is a consequence of the variational optimization of the wave function and is attributed to electron-photon correlation. Nevertheless, the photon contribution to the ground state is relatively small, even for large couplings. On the other hand, the excited states share a larger photon component since the electronic excitation is resonant with the cavity frequency. As a result, the electron-photon correlation is expected to be more relevant for the excited states, which should then be more stabilized than the ground state, as pictorially illustrated in Figure 1. Therefore, we expect electron-photon correlation in TD-SC-QED-HF to generally induce a redshift in the excitation energies compared to the TD-QED-HF results. The frequency redshift for TD-SC-QED-HF also contributes to the asymmetry in the Rabi splitting and, following eq 39, the LP intensity is generally larger for TD-SC-QED-HF than TD-QED-HF. The asymmetry of the spectrum also arises from the dipole self-energy, which effectively shifts the molecular electronic excitation, and from the contribution of higher energy states coupled to the cavity photon. The predicted redshifts of the excitation energies are more relevant for large coupling strengths λ, while the TD-QED-HF and TD-SC-QED-HF results are more similar for smaller couplings, as expected since both methods converge to the Hartree–Fock excitations (and the one-photon line) for λ → 0. For small light-matter couplings, the computed redshifts appear to be quadratic in λ, as we show in the Supporting Information. Since experimental setups rely on a large collective coupling, achieved with a relatively small single-molecule coupling λ and a large number of molecules coupled to the optical device, it is interesting to study whether such a redshift is also present in the collective regime. To this end, we focus on a smaller system, the hydrofluoric acid, described using the 6-31++g* basis set, to include more molecules in the simulation. In Figure 2, we report the lower (LP) and upper (UP) polaritonic excitation energies for *N* fluoridic acid molecules, with coupling strength λ such that $\lambda \sqrt{N}=$ 0.05a.u. for TD-QED-HF and TD-SC-QED-HF. To focus specifically on the effect of the electron-photon correlation, we also report the results computed by disregarding the DSE, which effectively renormalizes the electronic excitation and thus contributes to an effective cavity detuning. In Figure 2, we notice that the DSE has an appreciable effect on the excited states, even in the collective regime. We also notice that for QED-HF without the DSE, the upper and lower polariton energies do not change with *N*: the excitation energies depend only on the collective coupling strength $\lambda \sqrt{N}$ , in contrast to the TD-SC-QED-HF results which show λ dispersion even when the DSE is neglected due to the electron-photon correlation. In Figure 2, we see that the TD-SC-QED-HF energies are red-shifted compared to the TD-QED-HF excitations also in a collective-coupling setup, even though the differences are less relevant compared to the single-molecule case. The difference between the TD-QED-HF and TD-SC-QED-HF energies decreases with *N*, both including or disregarding the DSE, which suggests that the electron-photon correlation also depends on the single molecule coupling λ. Finally, we notice that the Rabi splitting is asymmetric even when the DSE is not included in the Hamiltonian for both methods in the single molecule and collective regimes (in contrast to what is expected from the two-level Jaynes–Cummings or Tavis–Cummings models). This is due to the nonperturbative nature of the *ab initio* QED approaches, which implicitly account for all the excited higher-energy states contributing to the polaritons.

**Table 3:** Ground State Energy *E*_GS_, Excitation Energy ω_*LP*_, Length Gauge Oscillator Strength *f*_*l*_, Photon Character ϑ_*LP*_, and Transition Photon Momentum *ip*_0*n*_ and Coordinate *q*_0*n*_ for the Lower Polariton (LP) of *p*-Nitroaniline (PNA) Computed Using the TD-QED-HF and TD-SC-QED-HF Models for Different Coupling Strengths λ Using the aug-cc-pvdz Basis Set^a^

λ [a.u.]	*E*_GS_ [a.u.]	ω_*LP*_ [a.u.]	*f*_*l*_	ϑ_*n*_	*q*_0*n*_ [a.u.]	*ip*_0*n*_ [a.u.]
TD-SC-QED-HF						
0.005	–489.27597426	0.178908	0.22644	0.5312	1.22775	0.21965
0.01	–489.23850592	0.175842	0.25831	0.5588	1.28009	0.22509
0.025	–489.26686246	0.165519	0.34288	0.6198	1.42221	0.23540
0.05	–489.27483426	0.146729	0.41351	0.6685	1.62482	0.23841
TD-QED-HF						
0.005	–489.27583031	0.178936	0.22418	0.5445	1.23350	0.22071
0.01	–489.27425941	0.175950	0.25341	0.5865	1.29089	0.22713
0.025	–489.26330961	0.166114	0.33003	0.6935	1.44337	0.23976
0.05	–489.22479115	0.148411	0.39369	0.8139	1.64752	0.24451

a The photon frequency is here set resonant to the first bright electronic excitation of PNA (with charge transfer character), and the polarization is along the transition dipole moment (along the C_2_ axis of PNA). The molecular geometry is reported in the Supporting Information.

**Figure 1:**
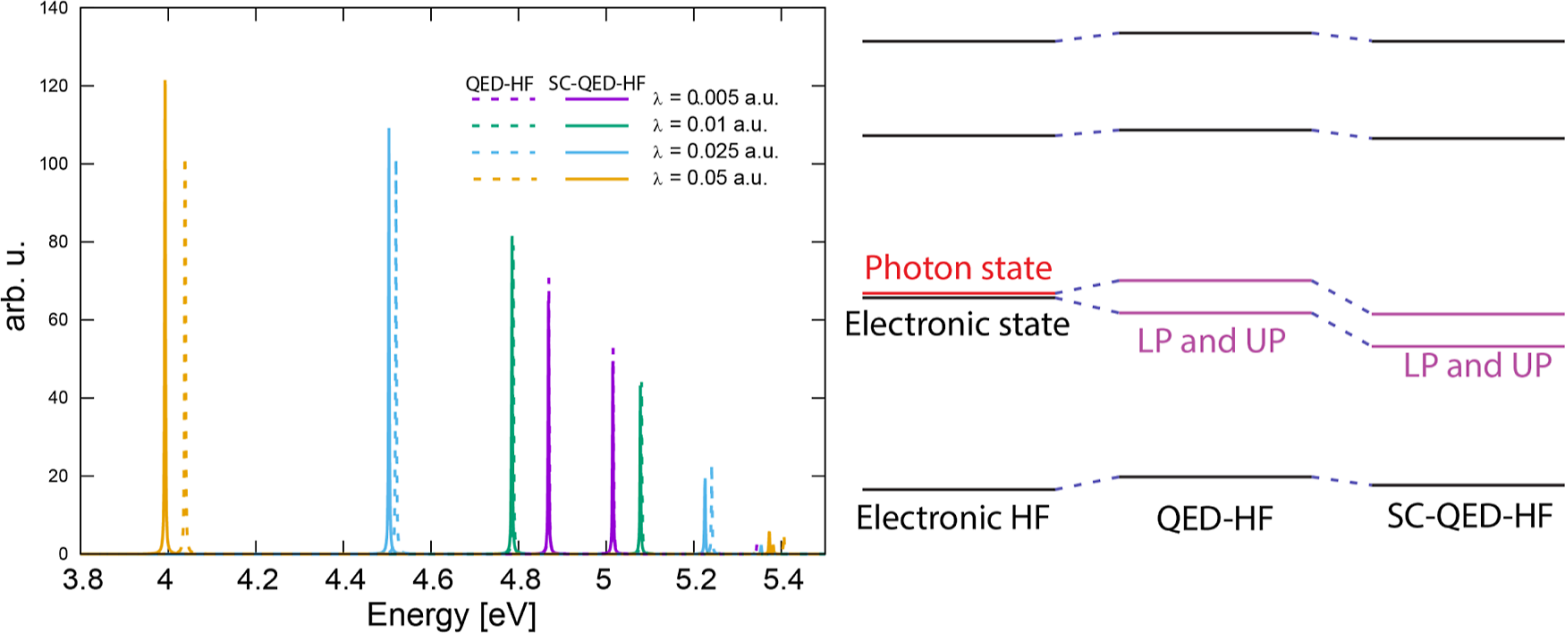
Left: absorption spectrum for the para nitroaniline (PNA) computed at TD-SC-QED-HF (solid lines) and TD-QED-HF (dashed lines) for different coupling strengths λ (each polaritonic excitation is endowed with a Lorentzian line shape). The polaritonic excitation energies of TD-SC-QED-HF are lower than the corresponding TD-QED-HF, as shown also in Table 3. Right: pictorial representation of the states in the QED-HF and SC-QED-HF models. Compared to the electronic HF results, the QED-HF states have higher energy levels due to the dipole self-energy contribution. Electron-photon correlation then stabilizes the SC-QED-HF states, compared to the mean-field QED-HF theory. Since the excited states share a larger photon component, the electron-photon correlation will be larger, thus leading to a more significant stabilization of the excited states compared to the ground state. As a result, the excitation energies present a redshift.

**Figure 2:**
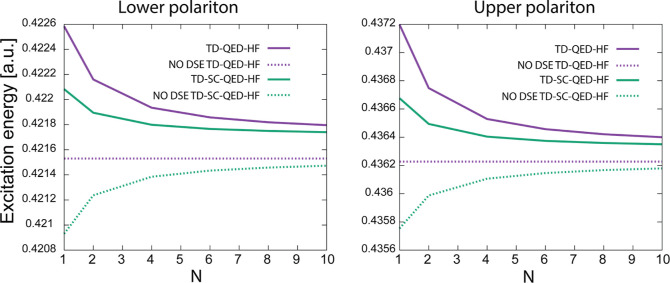
Lower polariton (left) and upper polariton (right) excitation energies computed using TD-QED-HF and TD-SC-QED-HF, including (solid lines) and disregarding (dotted lines) the dipole self-energy (DSE). We notice that the SC-QED-HF excitations are red-shifted compared to the mean field QED-HF energies due to the electron-photon correlation. For QED-HF, when the DSE is disregarded, the states depend only on the collective coupling strength $\lambda \sqrt{N}$ . In the collective regime, the TD-QED-HF and TD-SC-QED-HF energy differences are less pronounced than in the single-molecule simulations, which suggests that the electron-photon correlation depends mainly on the microscopic coupling strength λ.

## 4 Conclusions

In this paper, we have developed and implemented the linear response functions for the recently developed strong coupling quantum electrodynamics Hartree–Fock model (SC-QED-HF).^19^ The SC-QED-HF method is based on the exact ansatz in the infinite coupling limit, and the chosen time-dependent parametrization ensures that TD-SC-QED-HF recovers the exact excited state solutions for λ → ∞. At the same time, the (TD-)­SC-QED-HF model converges to (TD-)­QED-HF for small couplings. With the chosen time-dependent parametrization, the TD-QED-HF and TD-SC-QED-HF response equations have the same structure, the difference being only in the Hamiltonian transformation and the optimized molecular orbitals. We showed that for the developed TD-SC-QED-HF theory, the equivalence relations between the transition moments of photon and molecular observables are fulfilled, similarly to the TD-QED-HF model.^25,33^ In particular, the equivalence relation between dipole and velocity transition moments is fulfilled in a complete basis set (eq 26). Analogous relations hold for the photon coordinate and momentum (eq 28), but the relations involving the photonic boson operators hold exactly for any basis set, as demonstrated numerically in Section 3 (see the Supporting Information for the analytical proof). In Section 3, we explored the role of the dipole self-energy and compared the TD-SC-QED-HF results with the TD-QED-HF model. Since the SC-QED-HF ansatz introduces electron-photon correlation by explicitly mixing the electronic and electromagnetic degrees of freedom, comparing the SC-QED-HF and QED-HF results reveals the effect of electron-photon correlation in the ground and excited states. Our results suggest that electron-photon correlation induces a redshift in the polaritonic excitations compared to the mean field QED-HF results. However, our results for a collective-coupling ensemble suggest that electron-photon correlation is also connected to the microscopic light-matter coupling λ.

Our method provides another step in the development of *ab initio* QED methods based on the consistent SC-QED-HF wave function and provides an additional tool to analyze the effect of light-matter strong coupling on chemical properties. Since the SC-QED-HF convergence has been recently optimized by using second-order methods,^20^ future works will be devoted to the development of post-HF methodologies and higher-order response functions based on the more consistent SC-QED-HF orbitals.

## Supplementary Material



## Data Availability

The $e^{T}$ outputs are available in the following repository: DOI: 10.5281/zenodo.14577325. The code can be made available upon reasonable request to the authors.

## Footnotes

a The numerical values of the transition moments change with the basis set, but for each basis set the relation eq 31 is fulfilled.

## Notes

Additional results, further theoretical details and the molecular geometries employed in Section 3 (PDF)
