# Supplementary material for: Strong Coupling Quantum Electrodynamics Hartree–Fock Response Theory
Source: J Phys Chem A. 2025 May 9;129(20):4447–57. doi: 10.1021/acs.jpca.5c01166 (PMC12110736; doi:10.1021/acs.jpca.5c01166)
Supplement: Supplementary file 1 [file jp5c01166_si_001.pdf]

Supporting Information: Strong Coupling Quantum Electrodynamics Hartree-Fock Response Theory

Matteo Castagnola,[†] Rosario R. Riso,[†] Yassir El Moutaoukal,[†] Enrico Ronca,[‡] and Henrik Koch[*,†]

†*Department of Chemistry, Norwegian University of Science and Technology, 7491 Trondheim, Norway*

‡*Dipartimento di Chimica, Biologia e Biotecnologie, Università degli Studi di Perugia, Via Elce di Sotto, 8,06123, Perugia, Italy*

E-mail: henrik.koch@ntnu.no

1 Theory

In this section, we provide analytical proof that the TD-SC-QED-HF model fulfills the equivalence relations

$$\omega_n \langle 0|q|n\rangle = i \langle 0|p|n\rangle \tag{1}$$

and

$$\omega_n \langle 0|\boldsymbol{r}_i|n\rangle = i \langle 0|\boldsymbol{p}_i|n\rangle . \tag{2}$$

To this end, we prove that the first-order equation of motion[S1,S2]

$$\omega_1 \langle\langle A; V^{\omega_1}\rangle\rangle_{\omega_1} = \langle\langle [A, H]; V^{\omega_1}\rangle\rangle_{\omega_1} + \langle 0|[A, V^{\omega_1}]|0\rangle \tag{3}$$

is fulfilled.[S3] Consider the relation

$$iP = [Q, H], \tag{4}$$

and we want to prove that

$$\langle\langle P; V^{\omega_1}\rangle\rangle_{\omega_1} = -i\omega_1 \langle\langle Q; V^{\omega_1}\rangle\rangle_{\omega_1} + i \langle 0|[A, V^{\omega_1}]|0\rangle . \tag{5}$$

The response function for the operator P is

$$\begin{aligned}\langle\langle P, V\rangle\rangle_{\omega_1+i\epsilon} = &\frac{1}{\sqrt{2}}\sum_{ai}\kappa_{ai}^{\omega_1}\langle[[Q_X, H_X], E_{ai}]\rangle_R + \frac{1}{\sqrt{2}}\sum_{ai}\kappa_{ia}^{-\omega_1}\langle[[Q_X, H_X], E_{ia}]\rangle_R \\ &+\gamma^{\omega_1}\langle[[Q_X, H_X], b^\dagger]\rangle_R + \gamma^{-\omega_1}\langle[[Q_X, H_X], b]\rangle_R\end{aligned} \tag{6}$$

where $Q_X = U_X^\dagger Q U_X$ is the operator transformed with the ground state SC-QED-HF transformation, and the same is valid for the Hamiltonian H_X. The average value is then computed for the reference state

$$|R\rangle = |\text{HF}\rangle \otimes |0\rangle . \tag{7}$$

Notice that, for the electric dipole operator $Q = \boldsymbol{d}$ (for a complete basis set) and for the photon momentum p we have

$$\boldsymbol{d}_X = U_X^\dagger \boldsymbol{d} U_X = \boldsymbol{d} \qquad p_X = U_X^\dagger p U_X = p, \tag{8}$$

while for the photon coordinate

$$q_X = U_X^\dagger q U_X = \frac{b + b^\dagger}{\sqrt{2\omega}} + \frac{2}{\omega}\frac{1}{\sqrt{2\omega}}\sum_p \eta_p \tilde{E}_{pp} . \tag{9}$$

First, consider a one-electron operator Q

$$Q = \frac{1}{\sqrt{2}}\sum_{ai}(Q_{ai}E_{ai} + Q_{ai}^* E_{ia}) + \delta Q \tag{10}$$

where δQ includes only the redundant electronic operators E_{ab} and E_{ij}

$$\delta Q = \frac{1}{\sqrt{2}} \sum_{ab} Q_{ab} E_{ab} + \frac{1}{\sqrt{2}} \sum_{ij} Q_{ij} E_{ij} \tag{11}$$

where a and b refer to virtual orbitals while i and j to occupied oribtals in Equation 7. Taking Q to be a component of the electric dipole operator, we can substitute Q_X with Q in the response function (in the complete basis approximation) to obtain

$$\begin{aligned}
\langle\langle P, V \rangle\rangle_{\omega_1 + i\epsilon} =& \frac{1}{2} \sum_{aibj} \kappa_{ai}^{\omega_1} \left\langle [[Q_{bj} E_{bj} + Q_{bj}^* E_{jb}, H_X], E_{ai}] \right\rangle_R + \frac{1}{2} \sum_{aibj} \kappa_{ia}^{-\omega_1} \left\langle [[Q_{bj} E_{bj} + Q_{bj}^* E_{jb}, H_X], E_{ia}] \right\rangle_R \\
&+ \frac{1}{\sqrt{2}} \sum_{ai} \kappa_{ai}^{\omega_1} \left\langle [[\delta Q, H_X], E_{ai}] \right\rangle_R + \frac{1}{\sqrt{2}} \sum_{ai} \kappa_{ia}^{-\omega_1} \left\langle [[\delta Q, H_X], E_{ia}] \right\rangle_R \\
&+ \frac{1}{\sqrt{2}} \sum_{\alpha bj} \gamma_\alpha^{\omega_1} \left\langle [[Q_{bj} E_{bj} + Q_{bj}^* E_{jb}, H_X], b_\alpha^\dagger] \right\rangle_R + \frac{1}{\sqrt{2}} \sum_{\alpha bj} \gamma_\alpha^{-\omega_1} \left\langle [[Q_{bj} E_{bj} + Q_{bj}^* E_{jb}, H_X], b_\alpha] \right\rangle_R \\
&+ \sum_\alpha \gamma_\alpha^{\omega_1} \left\langle [[\delta Q, H_X], b_\alpha^\dagger] \right\rangle_R + \sum_\alpha \gamma_\alpha^{-\omega_1} \left\langle [[\delta Q, H_X], b_\alpha] \right\rangle_R \\
=& \begin{pmatrix} 0 & Q_{ai}^* & 0 & Q_{ai} \end{pmatrix} \begin{pmatrix} \boldsymbol{A} & \boldsymbol{B} \\ \boldsymbol{B}^* & \boldsymbol{A}^* \end{pmatrix} \begin{pmatrix} \gamma^{\omega_1} \\ \kappa_{ai}^{\omega_1} \\ \gamma^{-\omega_1} \\ \kappa_{ia}^{-\omega_1} \end{pmatrix} \\
&+ \frac{1}{\sqrt{2}} \sum_{ai} \kappa_{ai}^{\omega_1} \left\langle [[\delta Q, H_X], E_{ai}] \right\rangle_R + \frac{1}{\sqrt{2}} \sum_{ai} \kappa_{ia}^{-\omega_1} \left\langle [[\delta Q, H_X], E_{ia}] \right\rangle_R \\
&+ \sum_\alpha \gamma_\alpha^{\omega_1} \left\langle [[\delta Q, H_X], b_\alpha^\dagger] \right\rangle_R + \sum_\alpha \gamma_\alpha^{-\omega_1} \left\langle [[\delta Q, H_X], b_\alpha] \right\rangle_R
\end{aligned}$$

Now we can write, by using Jacobi's identity

$$
\begin{aligned}
&= \begin{pmatrix} 0 & Q_{ai}^* & 0 & Q_{ai} \end{pmatrix} \begin{pmatrix} \boldsymbol{A} & \boldsymbol{B} \\ \boldsymbol{B}^* & \boldsymbol{A}^* \end{pmatrix} \begin{pmatrix} \gamma^{\omega_1} \\ \kappa_{ai}^{\omega_1} \\ \gamma^{-\omega_1} \\ \kappa_{ia}^{-\omega_1} \end{pmatrix} \\
&- \sum_{ai} \kappa_{ai}^{\omega_1} \langle [[E_{ai}, \delta Q], H_X] \rangle_R - \sum_{ai} \kappa_{ai}^{-\omega_1} \langle [[H_X, E_{ai}], \delta Q] \rangle_R \\
&- \sum_{ai} \kappa_{ia}^{\omega_1} \langle [[E_{ia}, \delta Q], H_X] \rangle_R - \sum_{ai} \kappa_{ia}^{-\omega_1} \langle [[H_X, E_{ia}], \delta Q] \rangle_R \\
&- \sum_{\alpha} \gamma_{\alpha}^{\omega_1} \langle [[b_{\alpha}^{\dagger}, \delta Q], H_X] \rangle_R - \sum_{\alpha} \gamma_{\alpha}^{-\omega_1} \langle [[H_X, b_{\alpha}], \delta Q] \rangle_R \\
&- \sum_{\alpha} \gamma_{\alpha}^{\omega_1} \langle [[b_{\alpha}^{\dagger}, \delta Q], H_X] \rangle_R - \sum_{\alpha} \gamma_{\alpha}^{-\omega_1} \langle [[H_X, b_{\alpha}], \delta Q] \rangle_R \,.
\end{aligned}
$$

When δQ is applied on the reference eave function, the result is either zero or the same (rescaled) HF wave function, so the right commutators in the last four lines are identically zero. Then, δQ commutes with $b^{\dagger}$, so the last two lines are identically zero. Finally, if we compute the commutator between δO and E_{ai} or E_{ia} we get

$$
\begin{aligned}
[E_{lm}, E_{ai}] &= E_{li}\delta_{am} - E_{am}\delta_{il} \\
[E_{cd}, E_{ia}] &= E_{ca}\delta_{di} - E_{id}\delta_{ac}
\end{aligned}
\tag{12}
$$

so the first terms of the first two lines are zero either because of the deltas in 12 or because of the generalized Brillouin's theorem. We are thus left only with the first line, and from the

response equation we obtain

$$
\begin{aligned}
&= \begin{pmatrix} 0 & O_{ai}^* & 0 & O_{ai} \end{pmatrix} \left[i \begin{pmatrix} \langle [b, V^{\omega_1}] \rangle_R \\ \frac{1}{\sqrt{2}} \langle [E_{ja}, V^{\omega_1}] \rangle_R \\ \langle [b^\dagger, V^{\omega_1}] \rangle_R \\ \frac{1}{\sqrt{2}} \langle [E_{aj}, V^{\omega_1}] \rangle_R \end{pmatrix} + \omega_1 \begin{pmatrix} 1 & 0 & 0 & 0 \\ 0 & 1 & 0 & 0 \\ 0 & 0 & -1 & 0 \\ 0 & 0 & 0 & -1 \end{pmatrix} \begin{pmatrix} \gamma^{\omega_1} \\ \kappa_{ai}^{\omega_1} \\ \gamma^{-\omega_1} \\ \kappa_{ia}^{-\omega_1} \end{pmatrix} \right] \\
&= i \langle [O, V^{\omega_1}] \rangle_R + \omega_1 (O_{ia} \kappa_{ai}^{\omega_1} - O_{ai} \kappa_{ia}^{-\omega_1}) \\
&= i \langle [O, V^{\omega_1}] \rangle_R + \omega_1 \frac{1}{\sqrt{2}} \Big(\langle [O, E_{ai}] \rangle_R \kappa_{ai}^{\omega_1} + \langle [O, E_{ia}] \rangle_R \kappa_{ia}^{-\omega_1} \Big),
\end{aligned} \tag{13}
$$

which is precisely the equation of motion Equation 3 for the linear response. This relation ensures the equivalence between the dipole and velocity formulation for the transition moments

$$\omega_n \langle 0 | \boldsymbol{r}_i | n \rangle = i \langle 0 | \boldsymbol{p}_i | n \rangle . \tag{14}$$

A similar proof holds for the photon relation

$$\omega_n \langle 0 | q | n \rangle = i \langle 0 | p | n \rangle . \tag{15}$$

In this case, from Equation 9 we write

$$Q_X = \frac{1}{\sqrt{2}} \sum_{ai} (Q_{ai} E_{ai} + Q_{ai}^* E_{ia}) + \delta Q + \frac{b + b^\dagger}{\sqrt{2\omega}} \tag{16}$$

and obtain

$$
\begin{aligned}
\langle\langle P,V\rangle\rangle_{\omega_1+i\epsilon} =& \frac{1}{2}\sum_{aibj}\kappa_{ai}^{\omega_1}\left\langle[[Q_{bj}E_{bj}+Q_{bj}^{*}E_{jb}+\frac{b+b^{\dagger}}{\sqrt{\omega}},H_X],E_{ai}]\right\rangle_R \\
&+\frac{1}{2}\sum_{aibj}\kappa_{ia}^{-\omega_1}\left\langle[[Q_{bj}E_{bj}+Q_{bj}^{*}E_{jb}+\frac{b+b^{\dagger}}{\sqrt{\omega}},H_X],E_{ia}]\right\rangle_R \\
&+\frac{1}{\sqrt{2}}\sum_{ai}\kappa_{ai}^{\omega_1}\langle[[\delta Q,H_X],E_{ai}]\rangle_R+\frac{1}{\sqrt{2}}\sum_{ai}\kappa_{ia}^{-\omega_1}\langle[[\delta Q,H_X],E_{ia}]\rangle_R \\
&+\frac{1}{\sqrt{2}}\sum_{\alpha bj}\gamma_{\alpha}^{\omega_1}\left\langle[[Q_{bj}E_{bj}+Q_{bj}^{*}E_{jb}+\frac{b+b^{\dagger}}{\sqrt{\omega}},H_X],b_{\alpha}^{\dagger}]\right\rangle_R \\
&+\frac{1}{\sqrt{2}}\sum_{\alpha bj}\gamma_{\alpha}^{-\omega_1}\left\langle[[Q_{bj}E_{bj}+Q_{bj}^{*}E_{jb}+\frac{b+b^{\dagger}}{\sqrt{\omega}},H_X],b_{\alpha}]\right\rangle_R \\
&+\sum_{\alpha}\gamma_{\alpha}^{\omega_1}\langle[[\delta Q,H_X],b_{\alpha}^{\dagger}]\rangle_R+\sum_{\alpha}\gamma_{\alpha}^{-\omega_1}\langle[[\delta Q,H_X],b_{\alpha}]\rangle_R \\
=& \begin{pmatrix}\frac{1}{\sqrt{2\omega}} & Q_{ai}^{*} & \frac{1}{\sqrt{2\omega}} & Q_{ai}\end{pmatrix}\begin{pmatrix}\boldsymbol{A} & \boldsymbol{B}\\ \boldsymbol{B}^{*} & \boldsymbol{A}^{*}\end{pmatrix}\begin{pmatrix}\gamma^{\omega_1}\\ \kappa_{ai}^{\omega_1}\\ \gamma^{-\omega_1}\\ \kappa_{ia}^{-\omega_1}\end{pmatrix} \\
&+\frac{1}{\sqrt{2}}\sum_{ai}\kappa_{ai}^{\omega_1}\langle[[\delta Q,H_X],E_{ai}]\rangle_R+\frac{1}{\sqrt{2}}\sum_{ai}\kappa_{ia}^{-\omega_1}\langle[[\delta Q,H_X],E_{ia}]\rangle_R \\
&+\sum_{\alpha}\gamma_{\alpha}^{\omega_1}\langle[[\delta Q,H_X],b_{\alpha}^{\dagger}]\rangle_R+\sum_{\alpha}\gamma_{\alpha}^{-\omega_1}\langle[[\delta Q,H_X],b_{\alpha}]\rangle_R\,.
\end{aligned}
$$

Following the same reasoning as above, we obtain the same equation of motion for the linear response function, and thus the equivalence between the transition moments of the photon coordinate and momentum

$$\omega_n\langle 0|q|n\rangle = i\langle 0|p|n\rangle\,. \tag{17}$$

Notice that in this case, we do not need a complete basis set since the commutator relation

$$[b,b^{\dagger}]=1 \tag{18}$$

is fulfilled independently of the basis set size, and the equations are not solved in a truncated photon subspace space. The same proof clearly also holds for TD-QED-HF.[S3]

2 Additional results

In Table S1, we report the ground state energy E_{GS}, the excitation energy ω_n, the length gauge oscillator strength f_l, the photon character ϑ_n, and the photon momentum and coordinate transition moments for the lower (LP) and upper (UP) polaritonic states of p-nitroaniline (PNA) in the aug-cc-pVDZ basis set, computed using the TD-QED-HF and TD-SC-QED-HF models for different coupling strengths λ (see also Tab. 3 in the main text).

In Figure S1, we report the computed redshift of the TD-SC-QED-HF polaritons compared to TD-QED-HF as a function of the light-matter coupling strength λ for the system reported in Fig. 1 of the main manuscript. For small light-matter couplings, the redshift appears to be quadratic in the coupling strength, but a more complicated trend appears for large coupling strengths, in particular for the upper polaritonic branch.

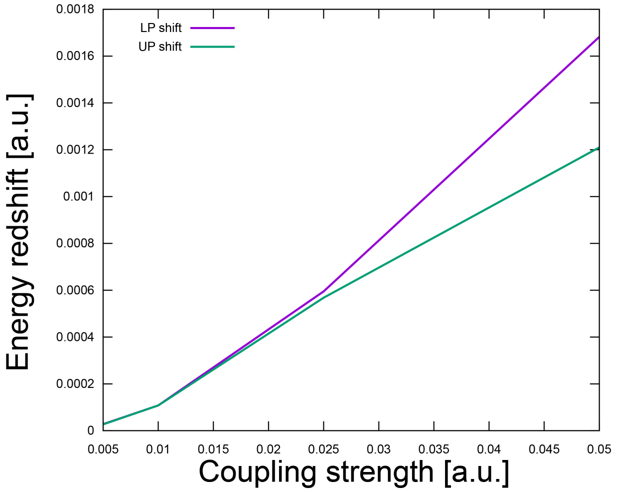

Figure S1: Redshift of the TD-SC-QED-HF polaritons compared to TD-QED-HF as a function of the light-matter coupling strength λ for the system reported in Fig. 1 of the main manuscript.

Table S1: Ground state energy E_{GS}, excitation energy ω_n, length gauge oscillator strength f_l, photon character ϑ_{LP}, the transition photon momentum ip_{0n} and coordinate q_{0n} for the lower polariton (LP) and upper polariton (UP) of the p-nitroaniline (PNA) computed using the TD-QED-HF and TD-SC-QED-HF models for different coupling strengths λ using the aug-cc-pVDZ basis set. The photon frequency is here set resonant to the first bright electronic excitation of PNA (with charge transfer character), and the polarization is along the transition dipole moment (along the C_2 axis of PNA). The molecular geometry is reported in the Supporting Information.

λ [a.u.]	E_{GS} [a.u.]	ω_n [a.u.]	f_l	ϑ_n	q_{0n} [a.u.]	ip_{0n} [a.u.]
Lower polariton (LP)						
TD-SC-QED-HF						
0.005	-489.27597426	0.178908	0.22644	0.5312	1.22775	0.21965
0.01	-489.23850592	0.175842	0.25831	0.5588	1.28009	0.22509
0.025	-489.26686246	0.165519	0.34288	0.6198	1.42221	0.23540
0.05	-489.27483426	0.146729	0.41351	0.6685	1.62482	0.23841
TD-QED-HF						
0.005	-489.27583031	0.178936	0.22418	0.5445	1.23350	0.22071
0.01	-489.27425941	0.175950	0.25341	0.5865	1.29089	0.22713
0.025	-489.26330961	0.166114	0.33003	0.6935	1.44337	0.23976
0.05	-489.22479115	0.148411	0.39369	0.8139	1.64752	0.24451
Upper polariton (UP)						
TD-SC-QED-HF						
0.005	-489.27597426	0.184307	0.16408	0.4657	1.11573	0.20563
0.01	-489.23850592	0.186614	0.13586	0.4294	1.05693	0.19723
0.025	-489.26686246	0.192037	0.07103	0.3189	0.87892	0.16878
0.05	-489.27483426	0.197437	0.02185	0.1764	0.62192	0.12279
TD-QED-HF						
0.005	-489.27583031	0.184334	0.16578	0.4537	1.10937	0.20449
0.01	-489.27425941	0.186722	0.13858	0.4068	1.04367	0.19487
0.025	-489.26330961	0.192605	0.07312	0.2737	0.84264	0.16229
0.05	-489.22479115	0.198647	0.01997	0.1198	0.54861	0.10898

3 Molecular Geometries

Table S2: Molecular geometry of the formaldehyde molecule. The coordinates are given in Angstrom Å

	Atom	x [Å]	y [Å]	z [Å]
1	H	-0.945037072500	0.000000000000	1.128390875700
2	C	0.000000000000	0.000000000000	0.526758766300
3	H	0.945037072500	0.000000000000	1.128390875700
4	O	0.000000000000	0.000000000000	-0.677166793600

Table S3: Molecular geometry of the para-nitroaniline molecule. The coordinates are given in Angstrom Å

	Atom	x [Å]	y [Å]	z [Å]
1	N	-2.157464580000	0.000000000000	0.000000000000
2	C	-0.703214720000	0.000000000000	0.000000000000
3	C	-0.011457210000	1.216931100000	0.000000000000
4	H	-0.567736250000	2.146975030000	0.000000000000
5	C	1.374820520000	1.216566830000	0.000000000000
6	H	1.914253020000	2.160555050000	0.000000000000
7	C	2.095469890000	0.000000000000	0.000000000000
8	N	3.466119360000	0.000000000000	0.000000000000
9	H	3.990101500000	0.861405000000	0.000000000000
10	H	3.990101500000	-0.861405000000	0.000000000000
11	C	1.374820520000	-1.216566830000	0.000000000000
12	H	1.914253020000	-2.160555050000	0.000000000000
13	C	-0.011457210000	-1.216931100000	0.000000000000
14	H	-0.567736250000	-2.146975030000	0.000000000000
15	O	-2.739659740000	1.091436200000	0.000000000000
16	O	-2.739659740000	-1.091436200000	0.000000000000

Table S4: Molecular geometry of the fluoridic acid. The coordinates are given in Angstrom Å

	Atom	x [Å]	y [Å]	z [Å]
1	H	0.000000000000	0.000000000000	0.000000000000
2	F	0.916800000000	0.000000000000	0.000000000000

References

(S1) Olsen, J.; Jørgensen, P. Linear and nonlinear response functions for an exact state and for an MCSCF state. *The Journal of chemical physics* **1985**, *82*, 3235–3264.

(S2) Dalgaard, E. Time-dependent multiconfigurational Hartree–Fock theory. *The Journal of Chemical Physics* **1980**, *72*, 816–823.

(S3) Castagnola, M.; Riso, R. R.; Barlini, A.; Ronca, E.; Koch, H. Polaritonic response theory for exact and approximate wave functions. *Wiley Interdisciplinary Reviews: Computational Molecular Science* **2024**, *14*, e1684.
